# Monoterpene-rich essential oil from *Artemisia santonicum* L. exerts neuroprotective effects in Aβ-induced SH-SY5Y cells: Modulation of tau pathology, neuroinflammation, oxidative stress, and synaptic-metabolic pathways

**DOI:** 10.1093/toxres/tfaf155

**Published:** 2025-11-12

**Authors:** Serap Nigdelioglu Dolanbay

**Affiliations:** Faculty of Science, Department of Biology, Gazi University, 06500, Teknikokullar, Ankara, Turkey

**Keywords:** Alzheimer’s disease, *Artemisia santonicum* L, monoterpene, neuroinflammation, oxidative stress, tau pathology

## Abstract

Understanding the complex biological mechanisms of ad requires innovative treatment approaches for this disease. In this context, natural compounds, especially monoterpenes, attract attention with their potential for biological activity. In this study, the therapeutic potential of monoterpene rich essential oil obtained from *Artemisia santonicum* L. for the treatment of ad was comprehensively evaluated. GC–MS analysis showed that the major monoterpenes were limonene, camphor, pinene, terpineol, and carvone in essential oil obtained from *A. santonicum* L. Possible common targets of monoterpenes with ad were predicted and their PPI networks were analyzed. Furthermore, gene set enrichment analysis was applied to understand the functional roles of these possible common targets and their relationships with biological pathways. Molecular docking studies revealed the binding affinities and interaction abilities of monoterpenes with the predicted possible common targets. The monoterpene rich essential oil obtained from *A. santonicum* L. used in our study provides a neuroprotective effect by targeting the pathological mechanisms of ad. We designed *in vitro* experiments to elucidate the mechanism of the mentioned neuroprotective effect. Within the scope of the study, neuroprotective effect analyses were performed to evaluate cell viability rates and *in vitro* AChE enzyme activity, while the ELISA method was used to determine phosphorylated tau levels and to assess neuroinflammatory responses. In addition, apoptosis levels, MMP changes and intracellular ROS accumulation were examined by flow cytometry analyses. These comprehensive analyses aimed to reveal the molecular mechanisms of the neuroprotective effect of monoterpene rich essential oil obtained from *A. santonicum* L. and to shed light on its potential therapeutic applications in ad.

## Introduction

Alzheimer’s disease (ad) is a neurodegenerative disorder characterized by abnormal aggregation and deposition of amyloid β (Aβ) peptides into extracellular plaques and hyperphosphorylated tau into intracellular neurofibrillary tangles. This leads to synaptic and neuronal loss, resulting in progressive cognitive and functional decline. ^1^ According to the World Alzheimer Report 2022, about 55 million individuals worldwide suffer from ad or related disorders, with that figure expected to rise to 82 million by 2030 and 138 million by 2050.^2^

In order to alleviate dementia symptoms, current dementia treatment focuses on acetylcholine and glutamate regulation. Among the treatments are acetylcholinesterase inhibitors (donepezil, galantamine, rivastigmine) and N-methyl-d-aspartate (NMDA) receptor antagonists (memantine).^3^^,^^4^ Nevertheless, there is currently no proven cure for ad; these drugs can only slow its course and alleviate its symptoms.^5^ In addition to existing dementia treatments, aducanumab, lecanemab, and donanemab have been approved by the FDA.^6^ However, there is ongoing research on the efficacy and safety of these drugs.

Due to the limitations of current synthetic drugs—such as limited efficacy, high cost, and potential side effects—there has been growing scientific interest in the identification of natural, multi-target agents with neuroprotective potential. In recent years, emphasis has been placed on finding anti-ad drug candidates derived from natural sources, which are thought to be safer and have fewer side effects than synthetic drugs.^7^ Monoterpenes are among the most biologically active compounds found in plants. Classified as plant secondary metabolites, monoterpenes exhibit a wide range of biological activities. They are frequently found as dominant components in the essential oils of species belonging to the *Artemisia* genus and hold a significant place in the chemical composition of this genus.^8–12^

Notably, monoterpenes have shown the ability to act on multiple pathological pathways implicated in ad, making them attractive candidates for multi-target therapeutic development. According to studies in the literature, monoterpenes such as limonene, camphor, pinene, terpineol, and carvone exhibit strong anti-Alzheimer effects in ad and offer a high level of neuroprotective potential by suppressing Aβ peptides, preventing tau protein phosphorylation, inhibiting cholinesterase activity, alleviating oxidative stress, inhibiting apoptosis, and suppressing inflammatory processes.^8^^,^^13–20^

This study hypothesizes that the essential oil of *Artemisia santonicum* L., rich in neuroactive monoterpenes, can target multiple interconnected ad-related pathological mechanisms — including tau hyperphosphorylation, oxidative stress, neuroinflammation, and apoptosis — and thereby exert a multi-target neuroprotective effect. In this context, *A. santonicum* L. was specifically selected for this study due to its high monoterpene content, its traditional use in the treatment of neurological disorders,^21^ and the fact that its anti-Alzheimer potential has not yet been extensively investigated in the literature.

In contrast to previous studies that have mostly focused on isolated monoterpenes or single-target synthetic drugs, this research emphasizes a holistic approach that leverages the synergistic effects of multiple active components. The novelty of this study lies in demonstrating that the essential oil of *A. santonicum* L., which contains a variety of monoterpenes, may exert synergistic effects on multiple pathological targets through multi-target mechanisms. In this respect, the study differs from previous research focused on isolated monoterpene compounds targeting a single pathway or synthetic drugs with limited activity profiles, and instead offers a more holistic and integrated therapeutic potential. Moreover, the proven neuroprotective properties of other *Artemisia* species in Alzheimer’s models^22–24^ support the consideration of *A. santonicum* L. as a promising and novel candidate in the context of ad.

In this study, the neuroprotective potential of the monoterpene-rich essential oil derived from *A. santonicum* L. was comprehensively evaluated in the context of ad. The research integrated computational and experimental approaches to investigate how the essential oil may interact with multiple ad-related pathological mechanisms, including AChE enzyme activity, tau phosphorylation, neuroinflammation, apoptosis, and oxidative stress. The findings suggest that *A. santonicum* L. essential oil may exert multi-target effects through key molecular pathways associated with ad, highlighting its potential as a promising natural therapeutic candidate. In order to robustly test this hypothesis, both *in silico* and *in vitro* approaches were employed in a complementary manner. Network pharmacology and molecular docking were used to systematically predict the potential targets and interactions of the monoterpene-rich essential oil, providing a theoretical foundation for its multi-target activity. These predictions were then validated through *in vitro* experiments, offering a comprehensive understanding of the oil’s neuroprotective mechanisms. This integrated strategy enhances both the reliability and the translational relevance of the findings.

## Materials and methods

### Plant material

*Artemisia santonicum* L. was collected from saline soils in August 2011 from Beypazarı, Ankara, Turkey. Plant taxonomy was confirmed by Prof. Dr Zeki Aytaç (Gazi University, Ankara, Turkey). Voucher specimen was deposited in the Gazi University Herbarium, Ankara, Turkey (ID: ZA-10434).

### Isolation of monoterpene rich essential oil

Dried samples of *A. santonicum* L. (500 g) were subjected to hydrodistillation for 4 hours using a Clevenger-type apparatus. The resulting monoterpene-rich essential oil was extracted with chloroform and subsequently dried over anhydrous sodium sulfate to eliminate residual moisture. The essential oil yield was calculated as approximately 0.85% (v/w) based on the dry weight of the plant material. The purity and composition of the oil were assessed by GC–MS analysis, which revealed that the monoterpene constituents accounted for approximately 73% of the total composition. Quality control measures included verification of consistent yields across three independent extractions, and assessment of physical parameters such as refractive index, color, and specific gravity in accordance with standard pharmacopoeial guidelines.

### GC–MS analysis

GC–MS analysis was performed with Agilent Technologies 6890 N Network GC-System 7683 B Serves Injector 5975 Inert MSD. The relative percentages of the monoterpenes were calculated from the total ion chromatogram (TIC). The relative retention indices (RRI) of the monoterpenes were calculated using the retention times of C8–C25 n-alkanes under the same chromatographic conditions (Table 1).

**Table 1:** QC summary of the GC–MS analysis.

**Parameter**	**Description**
Instrument	Agilent Technologies 6890 N GC-System with 7683B Injector and 5975 Inert MSD
Column Used	HP-5MS, 30 m × 0.25 mm, 0.25 μm
Carrier Gas	Helium, constant flow ~1 mL/min
Injection Mode	Split
Detection Mode	Electron impact (EI) ionization, 70 eV
Scan Range (m/z)	40–550 amu
Calibration Method	External standard calibration with C8–C25 n-alkane series
Retention Index Calculation	Relative Retention Indices (RRI) calculated based on n-alkanes (C8–C25)
Identification Criteria	Mass spectral match (≥90%) using NIST library + RRI comparison
Quantification Basis	Relative percentage calculated from Total Ion Chromatogram (TIC)
Reproducibility (RSD%)	<3% for major peaks, based on triplicate injections

### Prediction of possible common targets

Using SMILES data from PubChem, possible targets of 5 monoterpenes were predicted with Swiss Target Prediction web tool (http://www.swisstargetprediction.ch/). Possible targets of ad were predicted with DisGeNET web tool (https://disgenet.com/). Venn diagram showing possible common targets of monoterpenes and ad was drawn using Venny web tool (https://bioinfogp.cnb.csic.es/tools/venny/).

Swiss Target Prediction is an in silico tool based on the principle of molecular similarity. It compares the 2D and 3D structural features of a given small molecule to those of known bioactive compounds with experimentally validated targets. Based on this similarity, the tool predicts potential protein targets and assigns them probability scores. However, this approach may produce false negatives (failing to identify actual targets) or false positives (predicting incorrect targets), particularly for structurally unique or poorly represented compounds in the reference databases. Additionally, the accuracy of predictions is inherently limited by the scope and currency of the underlying datasets. DisGeNET, on the other hand, is a comprehensive platform focused on gene-disease associations. It integrates information from both manually curated literature sources and automatically mined data using text-mining algorithms. Consequently, while some gene-disease associations are supported by robust evidence, others may rely on limited or indirect data. This may lead to database bias or inconsistencies, and there may be significant data gaps for rare diseases or understudied genes.

### Protein–protein interaction (PPI) networks

PPI networks of all possible common targets were determined using STRING web tool (https://version11.string-db.org/). Then, the nodes and node degrees of all possible common targets have been determined. Finally, PPI networks of top 10 possible common targets were determined.

### Gene set enrichment analysis

As gene set enrichment analysis gene ontology (GO) and kyoto encyclopedia of genes and genomes analyzes (KEGG) were performed using the ShinyGO web tool (https://bioinformatics.sdstate.edu/go/).

### Molecular docking

Monoterpenes as ligands were downloaded from the PubChem in sdf format. Common possible targets (GAPDH, AKT1, ALB, PPARG, PTGS2, EGFR, MAPK3, HIF1A, ESR1, HSP90AA1, and AChE) associated with ad were downloaded from the RCDB PDB protein data bank (https://www.rcsb.org/) in pdb format. Cavity-detection guided blind docking of monoterpenes and possible common targets associated with ad was performed with CB-Dock2 (https://cadd.labshare.cn/cb-dock2/index.php).

### Cell treatments

SH-SY5Y cells were maintained in Dulbecco’s Modified Eagle Medium/Nutrient Mixture F-12 (DMEM/F-12; Gibco) supplemented with 10% fetal bovine serum (FBS; Gibco) and 1% penicillin–streptomycin at 37 °C in a humidified atmosphere of 5% CO₂. Cells between passages 5 and 15 were used in all experiments. Prior to treatment, cells were seeded at a density of 1 × 10^4^ cells/well in 96-well plates or 2 × 10^5^ cells/well in 6-well plates (Corning) and allowed to adhere for 24 h. For all experiments, non-differentiated SH-SY5Y cells were used. Aβ₁–₄₂ (Sigma-Aldrich) was dissolved in deionized water to a stock concentration of 100 μM and incubated at 37 °C for 24 h to promote oligomerization. The stock was then diluted to a working concentration of 10 μM immediately before use. Cells were divided into the following treatment groups:

Control group: Untreated cells.

Aβ group: Treated with 10 μM Aβ₁–₄₂ for 24 h.

Treatment groups: Pre-treated with monoterpene-rich essential oil from *Artemisia santonicum* L. (50, 100, 250, and 500 μg/mL) for 24 h, followed by co-treatment with 10 μM Aβ₁–₄₂ for an additional 24 h in the continued presence of the essential oil.

### MTT assays

Cell viability was determined using the thiazolyl blue tetrazolium bromide (MTT) assay. After treatment of monoterpene rich essential oil from *A. santonicum* L., all medium in the 96-well plates was removed and replace with medium containing 0.5 mg/mL MTT (Sigma-Aldrich). The plate was incubated at 37 °C for 4 h in 5% CO_2_ incubator, after which the MTT solution was removed and the cells were lysed with 200 μL of dimethyl sulfoxide (DMSO) (Sigma-Aldrich). The plate was then read at 570 nm using a microplate reader (Biotek Instruments). Absorbance values ​​were expressed as the percentage of untreated control cells (control = 100%).

### *In vitro* AChE enzyme activity

The *in vitro* acetylcholinesterase (AChE) inhibitory activity of monoterpene rich essential oil from *A. santonicum* L. was evaluated using a modified Ellman’s method. The assay was performed spectrophotometrically with acetylthiocholine iodide (Sigma-Aldrich) as the substrate and AChE enzyme (Sigma-Aldrich) derived from electric eel. Each reaction mixture contained 80 μL of phosphate buffer (pH 7, Merck), varying concentrations of monoterpene rich essential oil from *A. santonicum* L. (50, 100, 250, and 500 μg/mL), and 20 μL of AChE enzyme, followed by a 10-minute incubation at room temperature. The chromophore 5-thio-2-nitrobenzoic acid was generated by adding 5,5′-dithiobis(2-nitrobenzoic acid) (DTNB, Sigma-Aldrich). Subsequently, 50 μL of acetylthiocholine iodide was introduced, and the mixture was further incubated for 10 minutes. Absorbance was recorded at 412 nm.

### ELISA assays

Human Tau (Phospho) [pT181, pS199, pT231, and pS396], human PGE2, IL-6, TNF-α, and IL-10 levels were determined according to the user manual of the purchased kit (Invitrogen).

### Flow cytometry analysis

Apoptosis, mitochondrial membrane potential (MMP), and intracellular reactive oxygen species (ROS) rate (%) was determined according to the user manual of the purchased kit (Abcam and Cayman Chemical). For apoptosis analysis, annexin-V and propidium iodide (PI) were used, while 5,5′,6,6′-tetrachloro-1,1′,3,3′-tetraethylbenzimidazolylcarbocyanine iodide (JC-1) dye was employed to assess MMP, and 2′,7′-dichlorodihydrofluorescein diacetate (DCFDA/H2DCFDA) dye was utilized for ROS. Cells were analyzed using ACEA NovoCyte flow cytometry and ACEA NovoExpress software.

### Statistical analysis

Statistical analyses were performed using IBM SPSS Statistics version 21.0. Results are presented as mean ± standard deviation (SD). For comparisons between the untreated control group and the Aβ₁–₄₂-treated group, Levene’s test for homogeneity of variance was applied, followed by an independent samples t-test. To evaluate concentration- and/or time-dependent differences among groups treated with Aβ₁–₄₂ and varying doses of essential oil, one-way analysis of variance (ANOVA) was conducted. When significant differences were observed, Tukey’s Honestly Significant Difference (HSD) post hoc test was used for multiple comparisons. All experiments were performed in triplicate and repeated independently to ensure reproducibility. A *p*-value less than 0.05 was considered statistically significant.

## Results and discussion

GC–MS analysis revealed that the major monoterpenes in *Artemisia santonicum* essential oil were limonene, camphor, pinene, terpineol, and carvone (Table 2 and Supplementary Table 1). Similarly, Nikolova et al.^9^ reported limonene (2.7%), α-pinene (9.3%), β-pinene (15.2%), α-terpineol (9.2%), and carvone (0.8%), while Ferrante et al.^10^ found camphor (36.6%) and α-terpineol (0.5%). Variations in monoterpene type and concentration may result from environmental factors. Our findings align with those in the literature.

**Table 2:** The major monoterpene of essential oil obtained from *Artemisia santonicum* L. by GC–MS analysis.

**Monoterpenes**	**Relative area (%)**	**Relative retention index (RRI)**
1. Limonene	5.5	1187
2. Camphor	15.3	1143
3. α-Pinene	9.6	939
β-Pinene	10.3	979
4. α-Terpineol	9.4	1190
5. Carvone	2.5	1240

**Figure 1:**
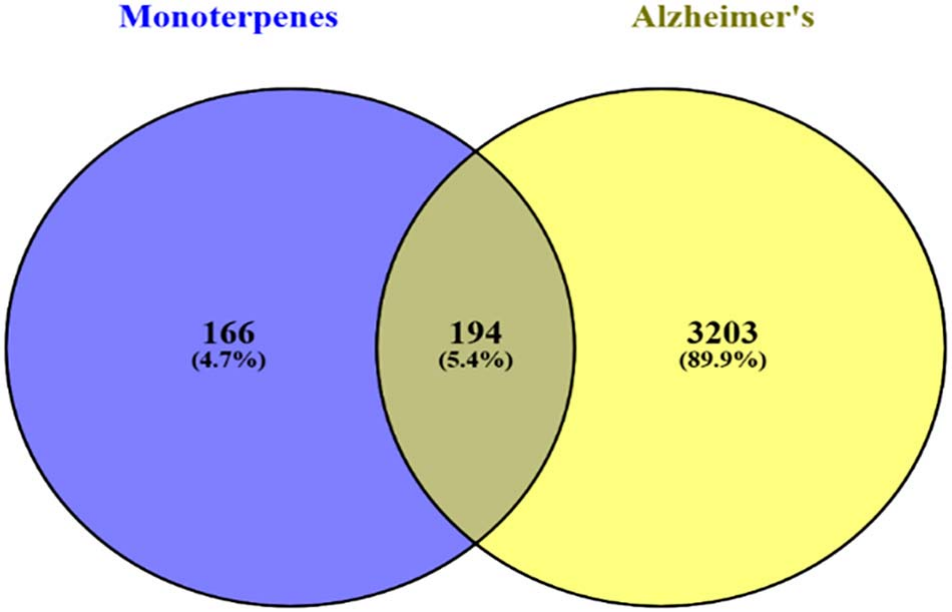
Venn diagram of possible common targets of monoterpenes and ad. Venn diagram illustrating the overlap between predicted targets of monoterpenes and ad-associated targets. The left circle represents 166 targets related only to monoterpenes (4.7%), the right circle represents 3203 targets associated only with ad (89.9%), and the overlapping area indicates 194 potential common targets (5.4%) shared by both monoterpenes and ad.

A Venn diagram illustrating potential common targets of monoterpenes and ad is shown in Fig. 1. Monoterpenes have 166 possible targets (4.7%), while ad has 3203 (89.9%). The overlap includes 194 common targets (5.4%), listed in Table 3.

**Table 3:** 194 possible common targets of monoterpenes and ad (grouped by function).

**Functional Group**	**Targets**
Receptors and neurotransmission	ADORA1, ADORA2A, ADRA1A, ADRA2A, ADRA2B, CHRM1, CHRM2, CHRM3, CHRNA7, DRD1, DRD2, DRD3, DRD4, HTR2C, HTR7, OPRD1, OPRK1, OPRM1, GABBR1, GRM5, GPBAR1, SLC18A3, SLC6A2, SLC6A3, SLC6A4, TRPA1, TRPV1
Enzymes	ACHE, ADA, ADH1B, ALDH1A1, ALDH2, ALOX12, ALOX5, CES1, CES2, CYP17A1, CYP19A1, CYP2C19, CYP2C9, DPP4, FAAH, GSR, HSD11B1, IDO1, MAOA, MAOB, MPO, NAT1, NAAA, PDE4D, PDE7A, PTGES, PTGS1, PTGS2
Kinases and signaling molecules	AKT1, CDK1, CHEK1, GSK3B, JAK2, MAP2, MAP2K1, MAP3K5, MAPK1, MAPK10, MAPK14, MAPK3, MAPK8, MAPK9, PRKCA, PRKCB, PRKCD, PRKCE, PTK2B, TGFBR1, TYK2
Transcription factors and epigenetic regulators	BRD2, BRD3, BRD4, HDAC1, HDAC3, HDAC6, HIF1A, NR1H2, NR1H3, NR1I2, NR1I3, NR3C1, NR3C2, PGR, PPARD, PPARG, RXRA, SIRT2, SREBF2
Transporters and structural proteins	ALB, ICAM1, SELE, SLC18A3, SLC6A2, SLC6A3, SLC6A4, TSPO, TTR
Immune and ınflammatory targets	CCR2, CCR5, CD38, CD81, CXCR2, CXCR3, IL6ST, NLRP3, TLR9
Other / miscellaneous	APP, AR, ASAH1, ATP12A, AHR, BACE1, BCHE, CPT1A, FABP3, FABP5, F13A1, FFAR1, G6PD, GAPDH, GBA, GLI1, GLI2, HMGCR, HMOX1, HPGDS, HSPA1A, IKBKB, KCNMA1, LIPA, LRRK2, MGLL, MPI, MTNR1A, MTNR1B, NPC1L1, NOS1, NOS2, NOS3, P2RX7, PARP1, PER2, PIN1, PLA2G1B, PLA2G4A, POLB, PREP, PTPN1, PTPN11, PTPRC, RBP4, RORA, SCD, SHBG, SIGMAR1, SPHK1, SPHK2, SQLE, TDO2, TERT, TGM2, TYR, VDR

**Figure 2:**
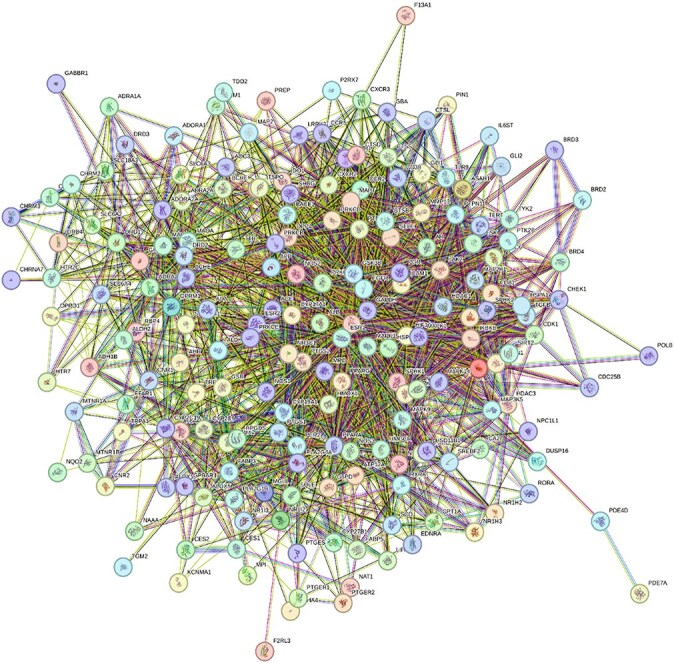
PPI network of 194 potential common targets shared between monoterpenes and ad. The network was constructed using the STRING database with a high-confidence interaction score threshold. Each node represents a protein, and each edge indicates a known or predicted interaction, including physical binding and functional associations. Node colors reflect clusters or functional groupings based on STRING’s evidence scoring. The dense connectivity of the network highlights the complex interplay among targets involved in ad-related pathways and suggests a multi-target mechanism of action for monoterpenes.

A PPI network illustrates protein interactions within a cell, helping to reveal functional relationships and disease mechanisms.^25^^,^^26^ The PPI network of 194 common targets of monoterpenes and ad (Fig. 2) includes 194 nodes and 2095 edges, with each node interacting with an average of 21.7 others—indicating dense connectivity. The average clustering coefficient is 0.523, suggesting frequent clustering. Compared to the expected 810 edges in a random network, the 2.5-fold increase highlights biologically meaningful interactions (*p* < 1.0e-16). Such dense connectivity may reflect the complex and multifactorial nature of ad, where numerous signaling pathways, metabolic processes, and cellular stress responses converge. The presence of highly clustered modules may indicate functional protein complexes or co-regulated pathways, many of which are involved in key ad-related mechanisms such as oxidative stress, inflammation, synaptic dysfunction, and tau pathology. The non-random structure of the network supports the hypothesis that the identified proteins—including those targeted by monoterpenes—play coordinated roles in ad progression. These findings reinforce the rationale for further exploring the interaction of monoterpenes within these tightly connected sub-networks, potentially as modulators of ad-related molecular pathways.

The 194 common targets of monoterpenes and ad were ranked by node degree. The top 10 targets were GAPDH (107), AKT1 (105), ALB (102), PPARG (84), PTGS2 (80), EGFR (74), MAPK3 (74), HIF1A (72), ESR1 (67), and HSP90AA1 (65).

**Figure 3:**
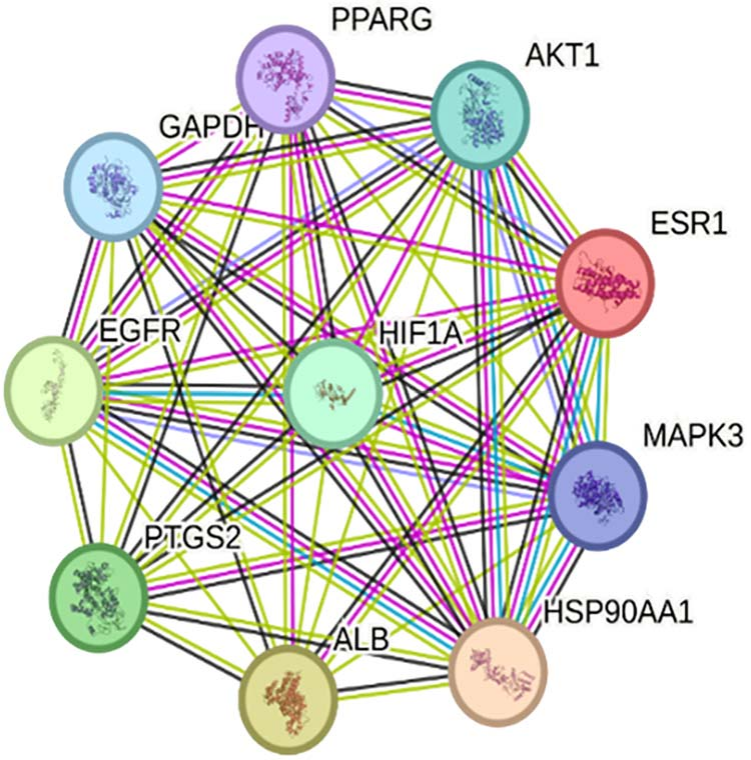
PPI network of the top 10 predicted common targets of monoterpenes and ad. The key hub proteins identified include AKT1, MAPK3, GAPDH, PTGS2, HSP90AA1, ALB, EGFR, ESR1, PPARG, and HIF1A. These proteins exhibit high connectivity and central positions within the network, suggesting their potential involvement in ad-related pathological pathways influenced by monoterpenes. The network was constructed using the STRING database with a high-confidence interaction score. Colored edges represent different types of evidence supporting the interactions, and node colors denote functional clusters or pathways.

The PPI network of the top 10 common targets of monoterpenes and ad (Fig. 3) includes 10 nodes and 45 edges, with an average of 9 interactions per node—indicating near-complete connectivity. The clustering coefficient is 1, showing full clustering. Compared to the expected 35 edges, the 1.3-fold increase suggests a denser-than-random network. The PPI enrichment *p*-value is 0.0194, indicating statistically meaningful, though modest, enrichment.

ad is a complex neurodegenerative disease involving various molecular mechanisms. GAPDH contributes to cell death via stress and apoptosis; AKT1 supports neuroprotection through signaling pathways. ALB reduces oxidative stress, while PPARG regulates inflammation and microglial activity. PTGS2 is linked to chronic inflammation in ad. EGFR influences cell growth, MAPK3 mediates stress responses and synaptic plasticity, and HIF1A responds to hypoxia. ESR1 promotes neuroprotection, and HSP90AA1 ensures proteostasis via protein folding. These targets help clarify ad mechanisms and offer potential therapeutic avenues. ^27–38^

GO and KEGG are key tools for analyzing gene functions. GO categorizes genes by biological process, cellular component, and molecular function, while KEGG maps genes and proteins to pathways involved in metabolism, signaling, and disease mechanism.^39–41^

**Figure 4:**
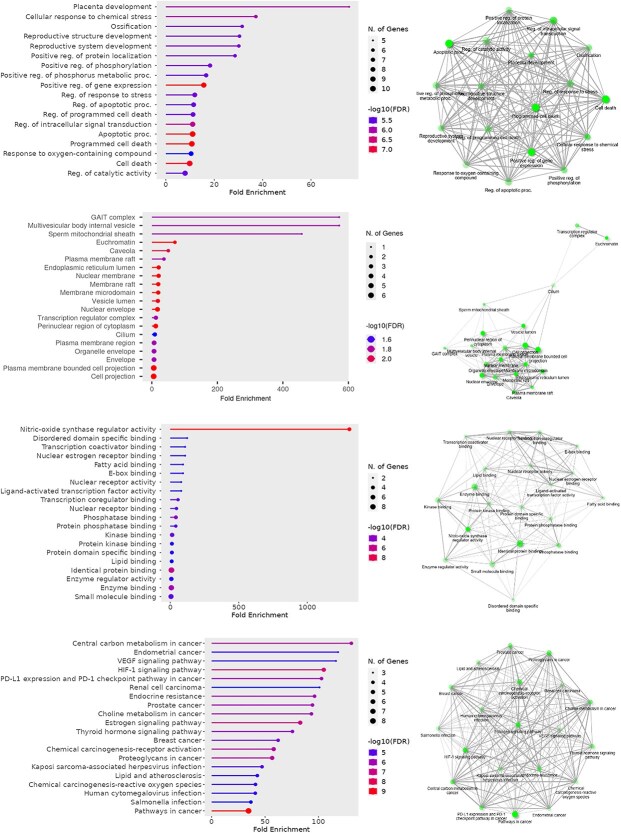
GO and KEGG enrichment analysis of the top 10 predicted common targets shared between monoterpenes and ad. The GO enrichment is categorized into three domains: Biological process (BP), cellular component (CC), and molecular function (MF), highlighting the key biological roles, subcellular localizations, and molecular activities associated with the selected targets. Additionally, KEGG pathway analysis reveals major signaling and metabolic pathways potentially involved in the neuroprotective effects of monoterpenes in ad. The figure summarizes the top enriched terms and pathways based on statistical significance (e.g. *p*-value or FDR).

GO biological process analysis of the top 10 common targets (Fig. 4) highlights roles in cellular stress response, oxidative balance, and regulation of cell death—key factors in ad progression. Processes like apoptosis, phosphorylation, and intracellular signaling are especially relevant due to their link to neurodegeneration and tau pathology. Regulation of oxygen-containing compound metabolism also reflects oxidative stress involvement in ad.^42–46^

GO cellular component analysis of the top 10 common targets (Fig. 4) highlights structures involved in protein folding, transport, and degradation. Disruptions in components like the GAIT complex, multivesicular bodies, and mitochondrial sheath contribute to beta-amyloid and tau accumulation in ad. Elements such as the ER lumen, caveolae, and plasma membrane rafts affect stress response and signaling, while the nuclear envelope and membrane microdomains influence gene regulation. Impairment of plasma membrane-bounded projections relates to synaptic dysfunction—key in ad pathology.^47–53^

GO molecular function analysis of the top 10 common targets (Fig. 4) reveals roles in neuroinflammation, oxidative stress, gene regulation, and metabolism. Nitric-oxide synthase regulation is linked to synaptic function; transcription and estrogen receptor binding relate to neuroprotection. Lipid and fatty acid binding affect membrane stability and amyloid accumulation. Protein kinase and enzyme regulator binding are tied to signaling and tau pathology. Small molecule and identical protein binding suggest therapeutic potential in ad.^54–60^

KEGG pathway analysis of the top 10 common targets (Fig. 4) highlights key ad-related mechanisms. HIF-1 and VEGF pathways relate to hypoxia and vascular dysfunction. PD-L1/PD-1 signaling is linked to neuroinflammation. Estrogen signaling and endocrine resistance reflect hormonal effects in ad. Metabolic pathways such as central carbon, choline, and lipid metabolism suggest energy imbalance and membrane instability. These pathways offer insight into the metabolic, inflammatory, and signaling disruptions in ad.^61–66^

CB-Dock2 is a web tool for molecular docking that predicts ligand-protein interactions by identifying binding pockets and calculating binding energies (vina scores).^67^ Vina scores obtained from molecular docking analyses represent the binding energy between a ligand and its target protein and are generally expressed as negative values in kcal/mol. These scores help predict the strength of the interaction and the stability of the resulting complex. In general, the more negative the score, the stronger the binding and the more stable the complex. Interactions with Vina scores between 0 and − 4 kcal/mol are considered weak and are usually biologically insignificant. Scores between −4 and − 6 kcal/mol indicate moderate binding potential, while values between −6 and − 8 kcal/mol suggest good binding affinity and the likelihood of a stable interaction. Scores of −8 kcal/mol or lower represent very strong and highly stable binding, and such ligands may hold significant potential in drug development.^68^^,^^69^  Table 4 shows binding energies of the top 10 common ad targets with monoterpenes. Target ranking: PTGS2 > AKT1 > ALB = HSP90AA1 > ESR1 > MAPK3 > PPARG > GAPDH > EGFR > HIF1A. Monoterpene ranking: Carvone > limonene > terpineol > pinene > camphor. Results indicate strong binding, suggesting therapeutic potential in ad.

**Table 4:** Binding energies (vina scores) of top 10 possible common targets of monoterpenes and ad.

**Monoterpenes**	**Possible Common Targets**									
	**GAPDH**	**AKT1**	**ALB**	**PPARG**	**PTGS2**	**EGFR**	**MAPK3**	**HIF1A**	**ESR1**	**HSP90AA1**
1. Limonene	−5.6	−6.5	−6.3	−5.6	−6.3	−5.4	−5.9	−4.7	−6.1	−7.0
2. Camphor	−5.8	−5.8	−6.1	**−6.1**	−5.7	−5.4	−5.5	−4.8	−5.9	−4.4
3. Pinene	−5.4	−5.9	−6.0	−5.6	−5.7	−5.4	−5.6	−4.7	−6.0	−5.6
4. Terpineol	−5.5	−5.6	−5.8	**−6.1**	−6.1	−5.6	−5.7	−4.7	**−6.5**	−6.0
5. Carvone	**−6.0**	**−6.6**	**−6.7**	−5.9	**−6.5**	**−5.8**	**−6.1**	**−4.9**	−6.2	**−7.3**

Table 5 shows the contact residues of the top-binding monoterpene for each of the top 10 common ad targets. Hydrogen bonds are marked with turquoise dotted lines, electrostatic interactions with yellow, and hydrophobic interactions with grey.^68^^,^^70^

**Table 5:** Contact residues of top 10 possible common targets of monoterpenes and ad.

**Monoterpenes**	**Possible** **Common** **Targets**	**Contact Residues**	
Carvone	GAPDH	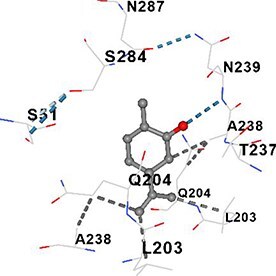	**Chain B:** SER51 THR52 PRO236 THR237 ALA238 ASN239 VAL240 SER284 ASN287 **Chain C:** LEU203 GLN204 **Chain D:** LEU203 GLN204 **Chain E:** SER51 THR52 PRO236 THR237 ALA238 ASN239 VAL240 SER283 SER284 ASN287
Carvone	AKT1	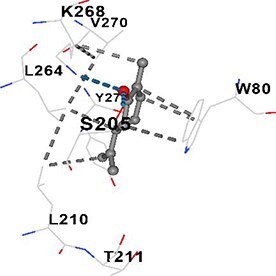	**Chain A:** ASN53 ASN54 GLN79 TRP80 SER205 LEU210 THR211 LEU264 LYS268 VAL270 VAL271 TYR272 ILE290 THR291 ASP292
Carvone	ALB	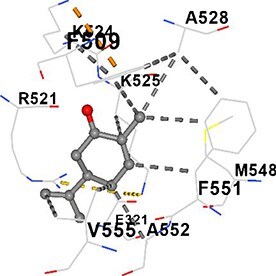	**Chain A:** LYS317 TYR319 ALA320 GLU321 LYS323 GLU358 CYS361 ALA362 **Chain B:** LEU398 GLY399 GLU400 TYR401 LYS402 PHE509 ILE513 GLU518 LYS519 ARG521 GLN522 LYS524 LYS525 ALA528 MET548 PHE551 ALA552 VAL555 GLU556
*Terpineol*, Camphor	PPARG	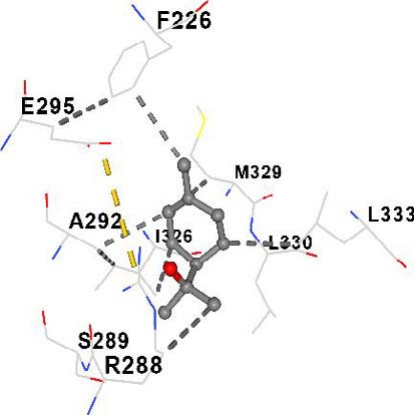	**Chain A:** ASP396 ARG443 **Chain B:** PHE226 PRO227 LEU228 ARG288 SER289 ALA292 GLU295 ILE296 LYS319 TYR320 HIS323 GLU324 ILE325 ILE326 MET329 LEU330 LEU333 GLU343 PRO366 GLU369 PHE370 LYS373 ARG397 PRO398 ASP441 LEU442 ARG443 GLN444 ILE445 VAL446 THR447 GLU448
Carvone	PTGS2	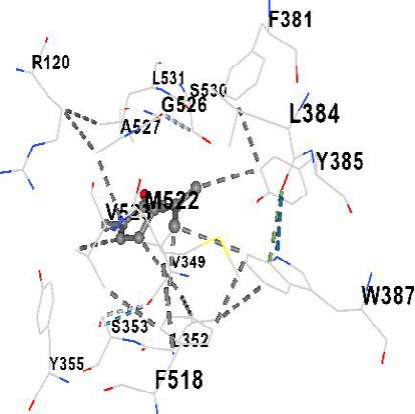	**Chain A:** LYS83 VAL89 HIS90 ILE92 LEU93 TRP100 ILE112 TYR115 VAL116 SER119 ARG120 TYR122 LEU123 VAL349 LEU352 SER353 TYR355 PHE381 LEU384 TYR385 TRP387 PHE470 SER471 ARG513 PHE518 MET522 VAL523 GLU524 GLY526 ALA527 PRO528 SER530 LEU531

*(Continued)*

**Table 5:** Continued.

**Monoterpenes**	**Possible** **Common** **Targets**	**Contact Residues**	
Carvone	EGFR	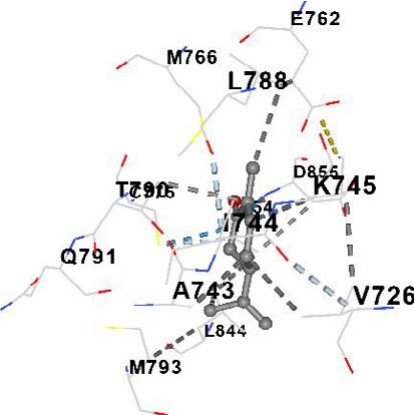	**Chain A:** LEU718 GLY719 VAL726 ALA743 ILE744 LYS745 GLU762 MET766 CYS775 LEU788 ILE789 THR790 GLN791 LEU792 MET793 GLY796 CYS797 ARG841 LEU844 THR854 ASP855
Carvone	MAPK3	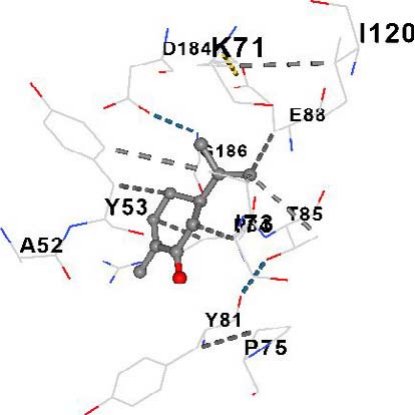	**Chain A:** ILE48 ALA52 TYR53 VAL56 ALA69 LYS71 ILE73 PRO75 TYR81 ARG84 THR85 GLU88 ILE101 ILE120 GLN122 ASP123 LEU124 MET125 GLU126 THR127 ASP128 LYS131 LEU173 CYS183 ASP184 GLY186
Carvone	HIF1A	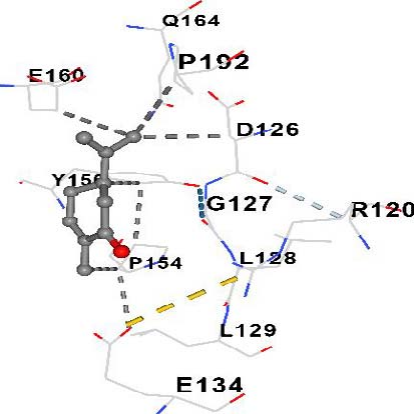	**Chain C:** ARG120 ASP126 GLY127 LEU128 LEU129 GLU134 PRO154 VAL155 TYR156 THR157 GLU160 GLN164 ASP190 HIS191 PRO192 ASN193 ASP197 ARG200 LEU201 GLU204
Terpineol	ESR1	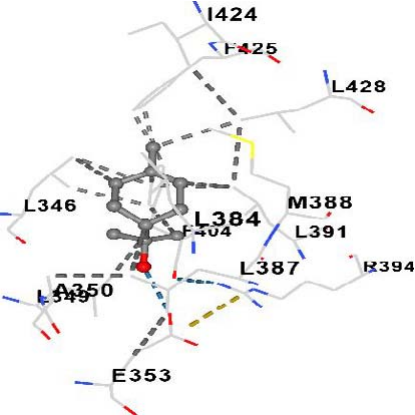	**Chain B:** MET343 LEU346 THR347 LEU349 ALA350 ASP351 GLU353 LEU354 TRP383 LEU384 LEU387 MET388 LEU391 ARG394 PHE404 ILE424 PHE425 LEU428 GLY521 MET522 LEU525 ASN532 VAL533 VAL534 PRO535 LEU539
Carvone	HSP90AA1	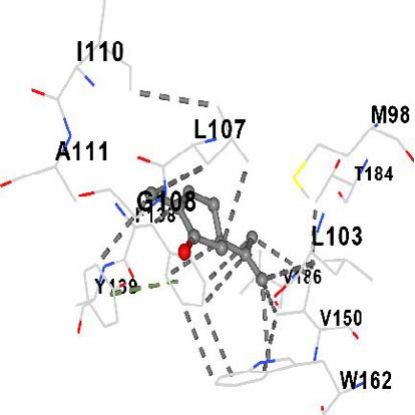	**Chain A:** PHE22 LEU48 ASN51 SER52 ALA55 ASP93 ILE96 GLY97 MET98 ASP102 LEU103 ILE104 LEU107 GLY108 ILE110 ALA111 GLY135 PHE138 TYR139 VAL150 TRP162 PHE170 THR184 VAL186

The 10 common targets of monoterpenes and ad are linked to Aβ/tau pathology (GAPDH, MAPK3, HSP90AA1), neuroinflammation (PPARG, PTGS2), oxidative stress/hypoxia (GAPDH, ALB, HIF1A), synaptic plasticity (AKT1, EGFR, ESR1), and metabolic dysfunction (GAPDH, HIF1A, PPARG).^71–81^ The monoterpene-rich *A. santonicum* essential oil may exert neuroprotective effects by modulating these pathways. In vitro experiments were designed to explore this mechanism.

SH-SY5Y cells treated with Aβ_1–42_ (10–100 μM, 24 h) showed decreased viability (*p* < 0.05), with 10 μM reducing viability to 42%. Monoterpene-rich essential oil from *A. santonicum* (50–500 μg/mL) restored viability to 57–82% (*p* < 0.05), indicating protection against Aβ_1–42_ toxicity. The essential oil alone showed no cytotoxicity. This is the first report of its protective effect in this model.

**Figure 5:**
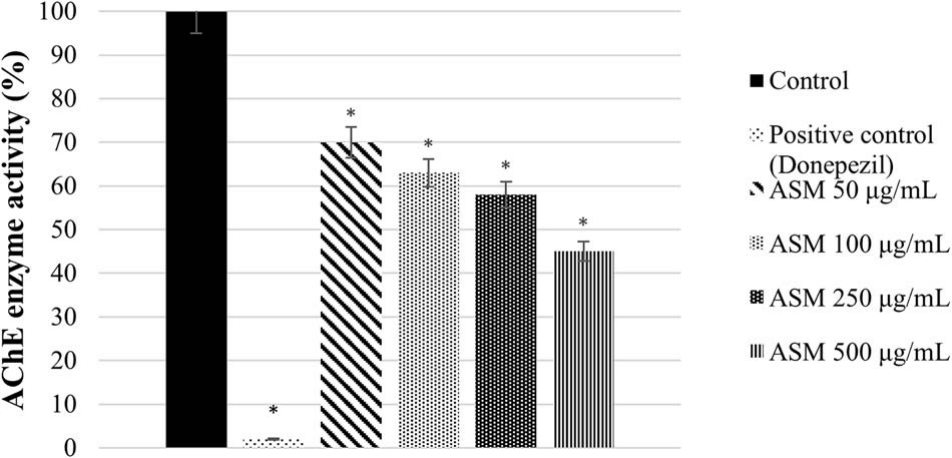
The inhibitor effect of monoterpene rich essential oil from *A*. *santonicum* L. on *in vitro* AChE enzyme activity. *p < 0.05 AChE enzyme activity (%) in groups compared to control. ASM: Monoterpene rich essential oil of *A*. *santonicum* L.

The monoterpene-rich essential oil obtained from *Artemisia santonicum* L. significantly inhibited electric eel AChE enzyme activity in a concentration-dependent manner. The highest inhibition was observed at a concentration of 500 μg/mL. Although this effect was not as strong as the positive control donepezil, it was still noteworthy. At lower concentrations (50 μg/mL), the inhibitory effect was more limited; however, a marked decrease in electric eel AChE enzyme activity was detected with increasing concentrations (Fig. 5).

Molecular docking analyses revealed that the monoterpenes present in the essential oil (limonene, camphor, pinene, terpineol, carvone) have a strong binding potential to the active site of the human AChE enzyme (Table 6). The docking scores (ranging from −6.4 to −6.6) were closely aligned, indicating similar affinities of the monoterpenes for the human AChE enzyme. Notably, pinene and carvone stood out with slightly lower energy scores (−6.6). Interactions were observed with key amino acid residues such as TRP86, TYR124, PHE295, TYR337, TYR341, and HIS447. These residues are located in the catalytic center and the peripheral anionic site of human AChE and play a critical role in the inhibition mechanism. These interactions may block the hydrolysis of acetylcholine, leading to its accumulation in the synaptic cleft and, consequently, an increase in neurotransmitter levels.

**Table 6:** Binding energies (vina scores) of AChE for monoterpenes.

**Monoterpenes**	**Vina Scores**	**Contact Residues**	
Limonene	−6.5	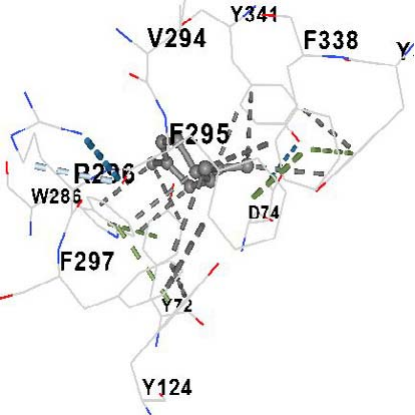	**Chain A:** TYR72 ASP74 LEU76 TYR124 TRP286 SER293 VAL294 PHE295 ARG296 PHE297 TYR337 PHE338 TYR341 **Chain B:** TYR72 ASP74 LEU76 TRP286 LEU289 SER293 VAL294 PHE295 TYR341
Camphor	−6.4	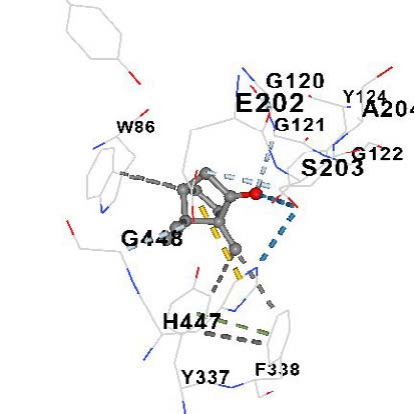	**Chain A:** TRP86 GLY120 GLY121 GLY122 TYR124 SER125 TYR133 GLU202 SER203 ALA204 TRP286 LEU289 SER293 VAL294 PHE295 ARG296 PHE297 TYR337 PHE338 TYR341 HIS447 GLY448 ILE451
Pinene	−6.6	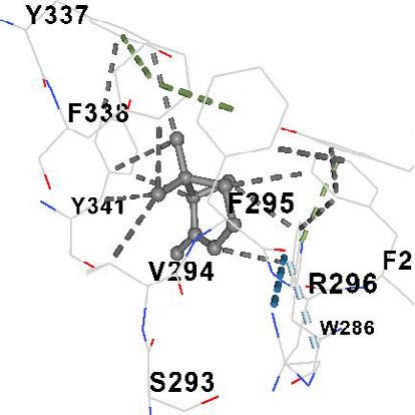	**Chain A:** TYR72 ASP74 LEU76 TRP286 SER293 VAL294 TYR341 **Chain B:** TYR72 ASP74 LEU76 TYR124 TRP286 LEU289 SER293 VAL294 PHE295 ARG296 PHE297 TYR337 PHE338 TYR341 GLY342
Terpineol	−6.5	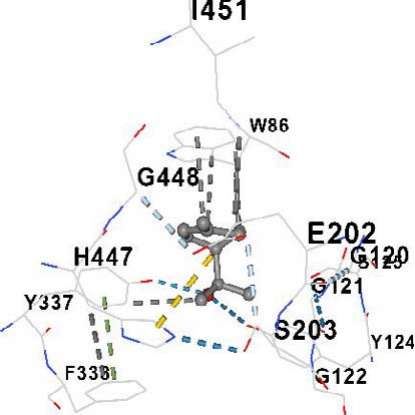	**Chain B:** TYR72 ASP74 LEU76 TRP86 GLY120 GLY121 GLY122 TYR124 SER125 GLY126 LEU130 TYR133 GLU202 SER203 ALA204 TRP286 LEU289 SER293 VAL294 PHE295 ARG296 PHE297 TYR337 PHE338 TYR341 GLY342 HIS447 GLY448 ILE451
Carvone	−6.6	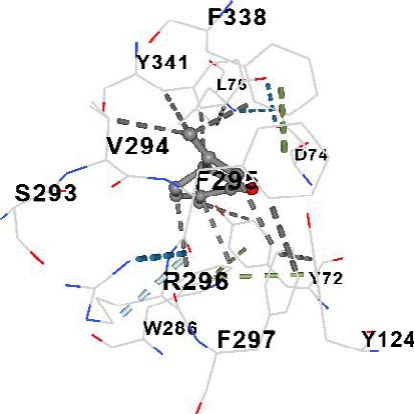	**Chain A:** TYR72 ASP74 LEU76 TRP86 GLY120 GLY121 GLY122 TYR124 SER125 GLY126 LEU130 TYR133 GLU202 SER203 TRP286 LEU289 SER293 VAL294 PHE295 ARG296 PHE297 TYR337 PHE338 TYR341 HIS447 GLY448 TYR449

The results suggest that the monoterpene-rich essential oil obtained from *A. santonicum* L. exhibits concentration-dependent inhibitory activity on electric eel AChE enzyme, and this effect may be attributed to its monoterpene content. In the literature, monoterpenes such as limonene, camphor, pinene, terpineol, and carvone have been reported to have protective effects on memory and cognitive functions.^19^^,^^82–85^

The IC_50_ values of essential oils from different *Artemisia* ecotypes for AChE inhibition range from 114 to 341 μg/mL.^86^ The AChE inhibition potential of essential oils from *Artemisia* species ranges between 29.7% and 55.2%. These values indicate that while the plants possess a certain level of inhibitory activity, their effect is lower compared to strong standard inhibitors such as huperzine A (90.7%) and galantamine (78.6%).^87^ Our findings are consistent with the literature. It can be concluded that while the monoterpene-rich essential oil obtained from *A. santonicum* L. is not as effective as the strongest inhibitors (donepezil, huperzine A, and galantamine), it presents a moderate level of AChE inhibitory potential among *Artemisia* species.

These findings suggest that the monoterpene-rich essential oil obtained from *A. santonicum* L., along with its constituent monoterpenes, may be promising candidates for supportive treatment of neurodegenerative diseases associated with cholinergic deficits, such as ad.

**Figure 6:**
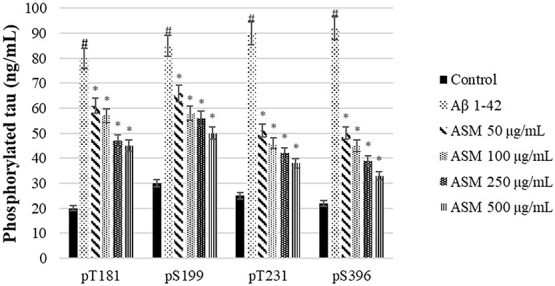
The suppressive effect of monoterpene rich essential oil from *A*. *santonicum* L. on phosphorylated tau levels (ng/mL) in SHSY-5Y cells. #*p* < 0.05 p-tau level in Aβ_1–42_-treated group compared to control; *p < 0.05 p-tau expression level in experimental groups compared to Aβ_1–42_-treated group. Aβ_1–42_: Amiloid beta_1–42_, ASM: Monoterpene rich essential oil of *A*. santonicum L.

**Figure 7:**
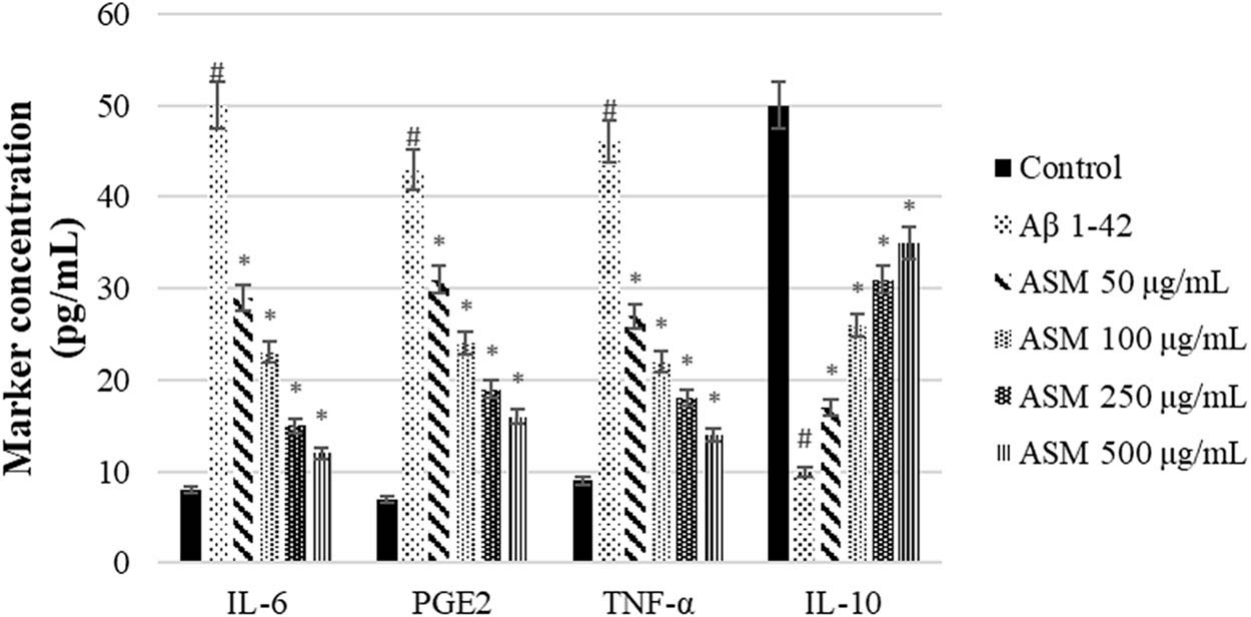
The regulator effect of monoterpene rich essential oil from *A*. *santonicum* L. on proinflammatory and anti-inflammatory marker concentrations (pg/mL) in SHSY-5Y cells. #*p* < 0.05 proinflammatory and antiinflammatory marker concentration in Aβ_1–42_-treated group compared to control; *p < 0.05 proinflammatory and anti-inflammatory marker concentration in experimental groups compared to Aβ_1–42_-treated group. Aβ_1–42_: Amiloid beta_1–42_, ASM: Monoterpene rich essential oil of *A*. *santonicum* L.

**Figure 8:**
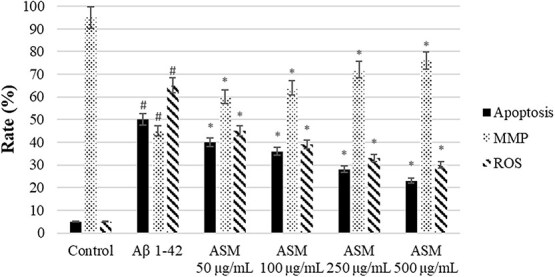
The regulator effect of monoterpene rich essential oil from *A*. *santonicum* L. on apoptosis, MMP, and intracellular ROS rate (%) in SHSY-5Y cells. #*p* < 0.05 rate (%) in Aβ_1–42_-treated group compared to control; *p < 0.05 rate (%) in experimental groups compared to Aβ*1–42*-treated group. Aβ*1–42*: Amiloid beta*1–42*, ASM: Monoterpene rich essential oil of *A*. *santonicum* L.

In this study, the neuroprotective effects of *A. santonicum* essential oil, rich in monoterpenes, were evaluated in SH-SY5Y cells exposed to Aβ₁–₄₂, a widely used in vitro model of ad. Our findings demonstrated that the essential oil significantly reduced levels of phosphorylated tau proteins (pT181, pS199, pT231, pS396), pro-inflammatory cytokines (IL-6, PGE₂, TNF-α), apoptosis, and ROS, while restoring anti-inflammatory IL-10 levels and MMP (Fig. 6-8). These results suggest a multifaceted protective effect of *A. santonicum* oil on ad-related cellular stress pathways.

Importantly, the oil’s effect on both early-stage (pT181, pT231) and late-stage (pS199, pS396) tau phosphorylation markers implies potential utility in both the prevention and treatment phases of ad.^88–92^ This is consistent with the findings of Shin et al.,^17^ who reported that six of sixteen terpenes derived from Korean forests, particularly limonene and pinene, reduced Aβ_1–42_-induced cell death, oxidative stress, and inflammation in a *Drosophila* model of ad. Furthermore, compounds such as carvone and terpineol, which are commonly found in monoterpene-rich essential oils, have previously been shown by Wojtunik-Kulesza et al.^19^ and Rawat et al.^93^ to possess AChE inhibitory activity, along with antioxidant effects and low cytotoxicity. These properties are of particular interest in the development of multi-target-directed ligands for ad. The presence of similar compounds in *A. santonicum* oil may underlie the observed neuroprotective effects in the current study.

Collectively, these results suggest that *A. santonicum* essential oil may offer therapeutic potential through simultaneous modulation of tau pathology, inflammation, oxidative stress, and mitochondrial dysfunction—hallmarks of ad pathology. The multifactorial action of the oil, possibly driven by bioactive terpenes such as carvone and terpineol, supports its further exploration as a candidate in the development of natural, multi-target therapies for ad. However, further in vivo studies and clinical evaluations are necessary to confirm these effects and determine therapeutic applicability.

The *in vitro* effects observed in SH-SY5Y cells align with the predicted targets and their associated GO/KEGG pathways. Reduced tau phosphorylation (pT181, pS199, pT231, pS396) may result from inhibition of MAPK3, GAPDH, and HSP90AA1—proteins linked to tau regulation and phosphorylation.^94–96^ Decreased ROS and increased mitochondrial membrane potential are consistent with modulation of GAPDH, ALB, and HIF1A, which maintain redox balance and mitochondrial integrity.^71^^,^^101^^,^^102^ Reduced IL-6, TNF-α, and PGE2, along with elevated IL-10, suggest anti-inflammatory effects mediated by PPARG and PTGS2. Docking confirmed strong monoterpene binding, particularly carvone, to PTGS2.^97–100^ In addition, synaptic plasticity and neuronal survival may be supported through modulation of AKT1, EGFR, and ESR1, which promote cellular resilience by inhibiting apoptosis.^103–105^ Metabolic and energy regulation is also likely maintained via GAPDH, HIF1A, and PPARG, which are essential for sustaining mitochondrial membrane potential and overall energy balance.^71^^,^^106^^,^^107^ These results support that monoterpenes from *A. santonicum* act on key targets regulating oxidative stress, inflammation, tau pathology, synaptic function, energy metabolism, and neuronal survival, confirming their neuroprotective potential. Table 7 presents the top binding monoterpenes for each protein target and their potential mechanistic effects. The essential oil likely exerts its neuroprotective effects by modulating these targets and pathways, as confirmed through integrated computational and in vitro analyses.

**Table 7:** Top binding monoterpenes per protein target and their potential mechanistic effects.

**Protein (Target)**	**Top Binding Monoterpene**	**Vina Score (kcal/mol)**	**Potential Mechanistic Effect** **(ad-related)**
GAPDH	Carvone	−6.0	Reduction of oxidative stress, decreased ROS, preservation of mitochondrial function
AKT1	Carvone	−6.6	Promotion of cell survival, inhibition of apoptosis, enhanced synaptic resilience
ALB	Carvone	−6.7	Antioxidant transport, redox balance, reduced Aβ aggregation
PPARG	Camphor / Terpineol	−6.1	Suppression of neuroinflammation, increased IL-10, regulation of microglial activity
PTGS2 (COX-2)	Carvone	−6.5	Inhibition of PGE2 synthesis, anti-inflammatory effect
EGFR	Carvone	−5.8	Support of neuronal growth and repair, improved cellular signaling
MAPK3 (ERK1)	Carvone	−6.1	Reduced tau phosphorylation, inhibition of neurotoxicity
HIF1A	Carvone	−4.9	Regulation of hypoxia responses, energy balance, mitochondrial protection
ESR1 (ERα)	Terpineol	−6.5	Estrogen-mediated neuroprotection, activation of antioxidant gene expression
HSP90AA1	Carvone	−7.3	Prevention of tau misfolding, maintenance of proteostasis

## Conclusions

In conclusion, 5 monoterpenes including limonene, camphor, pinene, terpineol, and carvone from *Artemia santonicum* L. were comprehensively evaluated for their potential use as therapeutic agents in ad. The associations of monoterpenes with ad-specific biological mechanisms were elucidated by possible common target prediction, PPI network analysis and gene set enrichment (GO and KEGG) methods. Molecular docking studies revealed the ability of these monoterpenes to interact with possible common targets (GAPDH, AKT1, ALB, PPARG, PTGS2, EGFR, MAPK3, HIF1A, ESR1, HSP90AA1, and AChE) and their binding energies. This study demonstrated that the monoterpene rich essential oil obtained from *A. santonicum* L. exerts neuroprotective effects by targeting key pathological mechanisms in ad. Through *in vitro* experiments, the underlying mechanisms of this effect were elucidated, showing that the monoterpene rich essential oil obtained from *A. santonicum* L. supports cell viability, inhibits electric eel AChE enzyme activity, reduces phosphorylated tau levels, and modulates neuroinflammatory responses. Furthermore, it was observed that the monoterpene rich essential oil obtained from *A. santonicum* L. mitigates apoptosis, preserves MMP, and decreases intracellular ROS accumulation, as evidenced by flow cytometry analyses. These findings suggest that the monoterpene rich essential oil from *A. santonicum* L. holds promise as a potential therapeutic agent for ad and warrants further investigation in preclinical and clinical settings.

## Informed consent statement

Not applicable.

## Supplementary Material

Supplementary_table_1_tfaf155

## Data Availability

The original contributions presented in the study are included in the article. Further inquiries can be directed to the corresponding author.
