# Supplementary material for: Monoterpene-rich essential oil from Artemisia santonicum L. exerts neuroprotective effects in Aβ-induced SH-SY5Y cells: Modulation of tau pathology, neuroinflammation, oxidative stress, and synaptic-metabolic pathways
Source: Toxicol Res (Camb). 2025 Nov 12;14(6):tfaf155. doi: 10.1093/toxres/tfaf155 (PMC12608079; doi:10.1093/toxres/tfaf155)
Supplement: Supplementary_table_1_tfaf155 [file supplementary_table_1_tfaf155.docx]

**Supplementary table 1.** *A. santonicum* L. essential oil composition

| **No** | **Compound name** | **Relative area (%)** | **Relative retention index (RRI)** |
| --- | --- | --- | --- |
| 1 | α-Thujene | 0.8 | 924 |
| 2 | α-Pinene | 9.6 | 939 |
| 3 | Camphene | 1.2 | 954 |
| 4 | β-Pinene | 10.3 | 979 |
| 5 | Sabinene | 1.0 | 983 |
| 6 | Myrcene | 0.7 | 991 |
| 7 | α-Phellandrene | 0.4 | 1005 |
| 8 | 1,8-Cineole (Eucalyptol) | 1.7 | 1033 |
| 9 | Limonene | 5.5 | 1187 |
| 10 | Terpinolene | 0.6 | 1089 |
| 11 | Camphor | 15.3 | 1143 |
| 12 | Borneol | 1.8 | 1165 |
| 13 | α-Terpineol | 9.4 | 1190 |
| 14 | Carvone | 2.5 | 1240 |
| 15 | Caryophyllene | 0.9 | 1418 |
| 16 | Germacrene D | 1.1 | 1480 |
| 17 | Spathulenol | 0.5 | 1577 |
